# Going beyond participation: community-led design and evaluation of the Philly Joy Bank

**DOI:** 10.3389/fpubh.2026.1725812

**Published:** 2026-03-04

**Authors:** Allison K. Groves, Nia Coaxum, Ayomide Sokale, Yuan He, Stacey Kallem, Chaquita Calloway, Nora L. Lee, Erikka Gilliam, Jordan S. Wilson, Daniel T. Vader, Nia Samuels, Imani Davis, Ariel Presley, Christina Brown, Rita Nelson, Hyden Terrell, George Dalembert, Cara Frances, Olivia Cordingley, Elizabeth S. Valdez

**Affiliations:** 1 Drexel University Dornsife School of Public Health, Philadelphia, PA, United States; 2 Philadelphia Department of Public Health, Philadelphia, PA, United States; 3 The Children's Hospital of Philadelphia Department of Pediatrics, Philadelphia, PA, United States; 4 Philadelphia Community Action Network, Philadelphia, PA, United States

**Keywords:** birth outcomes, cash transfers, community-based participatory research (CBPR), mental health, mixed methods, photovoice, postpartum, pregnancy

## Abstract

**Introduction:**

In Philadelphia there are racial disparities in health outcomes during the perinatal period, such that Black infants and their parents experience a disproportionately higher burden of poor outcomes compared to their White counterparts. These excess risks are driven at least in part by high rates of poverty and other manifestations of structural racism. The provision of guaranteed income (GI) or unconditional cash payments during and after pregnancy, is a bold and evidence-based approach to advancing equity in financial security and health. The objective of this protocol paper is to describe the community-centered approach to the design of (a) the Philly Joy Bank (PJB), a perinatal GI program in Philadelphia, and (b) the evaluation of PJB’s impact.

**Methods:**

PJB was conceived by an established coalition of community partners through a collective impact model. Decisions related to the design and implementation of PJB are made through a consensus building process that centers the voices of Lived Experience Experts (i.e., Black birthing people in Philadelphia). Congruent with the community-driven design of PJB, the evaluation of the impact of PJB is grounded in the principles of community-based participatory research and is co-designed by the community.

**Results:**

The study purpose is to assess the feasibility and acceptability of PJB (Aim 1); to examine *whether* and *how* GI affects ability to meet basic needs and parental stress (Aim 2); and to explore the impact of PJB on parental mental health and the preliminary impact on infant prematurity (low birthweight and preterm birth) (Aim 3). Descriptive statistics, mixed effects regression analyses, and participatory qualitative analysis approaches will be used to achieve study aims.

**Discussion:**

GI is a promising upstream structural intervention to address persistent health inequities during the perinatal period: a critical period of the life course. Understanding if and how GI improves health for birthing people and their infants can inform implementation and policy to advance health equity.

## Introduction

Adverse birth outcomes—including preterm birth, low birthweight, and infant mortality—remain significant public health challenges in Philadelphia and across the United States (U.S.). In both contexts, Black infants experience a disproportionately higher burden of poor outcomes, with rates of preterm birth (1, 2), low birthweight (3), and infant mortality (2, 4) consistently about twice those observed among White infants. Birthing parents also experience stark racial inequities in both physical and mental health outcomes. Commonly cited adverse outcomes include perinatal depression and maternal mortality. In Philadelphia and across the U.S., rates of perinatal depression are approximately 60% higher among Black individuals than among their white counterparts (5, 6). The overall maternal mortality rate in Philadelphia, 20 deaths per 100,000 live births, is approximately 15% higher than the national average of 17.4 deaths per 100,000 live births (7–9). Furthermore, within the city, Black birthing people are four times more likely to die from pregnancy-related causes than white birthing people (7).

Financial insecurity often increases around the time of childbirth, with household income dropping by up to 10% during the months surrounding pregnancy (10), which can negatively impact health. Financial insecurity in the U.S. is rooted in a history of structural racism that has systematically limited access to vital resources for racialized groups (11–13). As a result, 40% of Black and Hispanic birthing people experience poverty in the perinatal months, even after accounting for government support (14).

Guaranteed income (GI) is a social policy intervention designed to reduce financial insecurity by providing cash transfers on a regular basis to certain populations, like birthing parents. In GI programs, cash transfers are unconditional: there are no work requirements tied to receiving the money and there are no conditions as to how the money can be spent. This approach has the potential to positively impact the health of both birthing parents and infants during and after pregnancy. GI programs reduce poverty-related stress by increasing an individual’s ability to direct cash toward their basic needs (15, 16). The number of perinatal GI programs in the U.S. has grown tremendously, such that at least 39 pilot programs are currently underway (17). Despite the promise of GI as a potential intervention to reduce health inequities during and after childbirth, there is limited guidance on the use of community-centered approaches to design and evaluate perinatal GI programs.

The objective of this protocol paper is to describe the community-centered approach to the design of the (a) Philly Joy Bank (a perinatal GI program in Philadelphia), and (b) the mixed- methods longitudinal evaluation of Philly Joy Bank’s impact on infant and parent health outcomes.

## Methods and analysis

### Part 1. Development of the PJB program

#### Community conceptualization

The Philly Joy Bank (PJB) was conceptualized and originated from the Holistic Mental Health (HMH) workgroup of the Philadelphia Community Action Network (CAN). The CAN is a multi-sector collective impact coalition (18) of maternal and child health stakeholders, which includes lived experience experts (LEEs), or community members who identify as a Black birthing person or parent. The primary goal of the CAN is to eliminate racial disparities in infant mortality in Philadelphia. The Division of Reproductive, Adolescent, and Child Health (ReACH) within the Philadelphia Department of Public Health is the backbone coordinating entity for the CAN. The CAN is rooted in community voices and leadership. Each workgroup is co-chaired by a LEE and uses a consensus-building model to make decisions. LEE voices are centered in the process in two ways: (a) after each major decision is presented and discussed, space is created for LEEs to share their perspectives; and (b) to promote equity and flatten hierarchies of power, LEE votes are counted twice.

In 2021, the CAN led a strategic planning process that identified financial stress as a key driver of poor birth outcomes among Black birthing parents in Philadelphia. The CAN employed the use of a perinatal periods of risk (PPOR) analysis (19) to identify factors that impact poor birth outcomes and infant mortality. With inspiration from other maternal and infant-focused GI groups such as the Abundant Birth Project (San Francisco) and the Bridge Project (New York City), the HMH workgroup selected perinatal GI as a strategy to reduce financial stress for birthing parents and their families in Philadelphia.

#### Community involvement in formative research

The Philadelphia CAN utilizes a collective impact model where initiatives have a shared purpose, are community-focused, community-driven, and collaborative. In line with these principles, the design of Philly Joy Bank was led by LEEs, and involved input from diverse stakeholders across Philadelphia and the U.S. A formal consulting relationship was established with the Abundant Birth Project, a GI program in San Francisco, to help guide initial program design. Based on their recommendations, local policy researchers conducted formative research—including interviews and focus groups—with both policy stakeholders (*n* = 4) and community stakeholders (*n* = 9). The goal was to guide the design, implementation, and sustainability of the program. Community participants were specifically limited to individuals who were either pregnant (*n* = 2) or parents of a child under 1 year old (*n* = 7).

The HMH workgroup held a planning retreat to share and incorporate findings from the formative research. Key recommendations included: (a) the alignment of the length of the program with existing maternal and infant health programs, with most recommending that the program last until 12 months postpartum; (b) a larger benefit amount informed by a family needs calculator to account for rising costs of living, and lived experience indicating that existing public benefits do not adequately meet needs; (c) that eligibility should not be limited only to those with the lowest income, both to account for those who do not qualify for public benefits yet still struggle to meet basic needs and to avoid the indignity of having participants prove that they are “poor enough” to qualify, and (d) the inclusion of individualized benefits counseling and other mitigation measures to reduce the risk of losing access to existing public benefits.

#### Conceptual model

**Figure 1:**
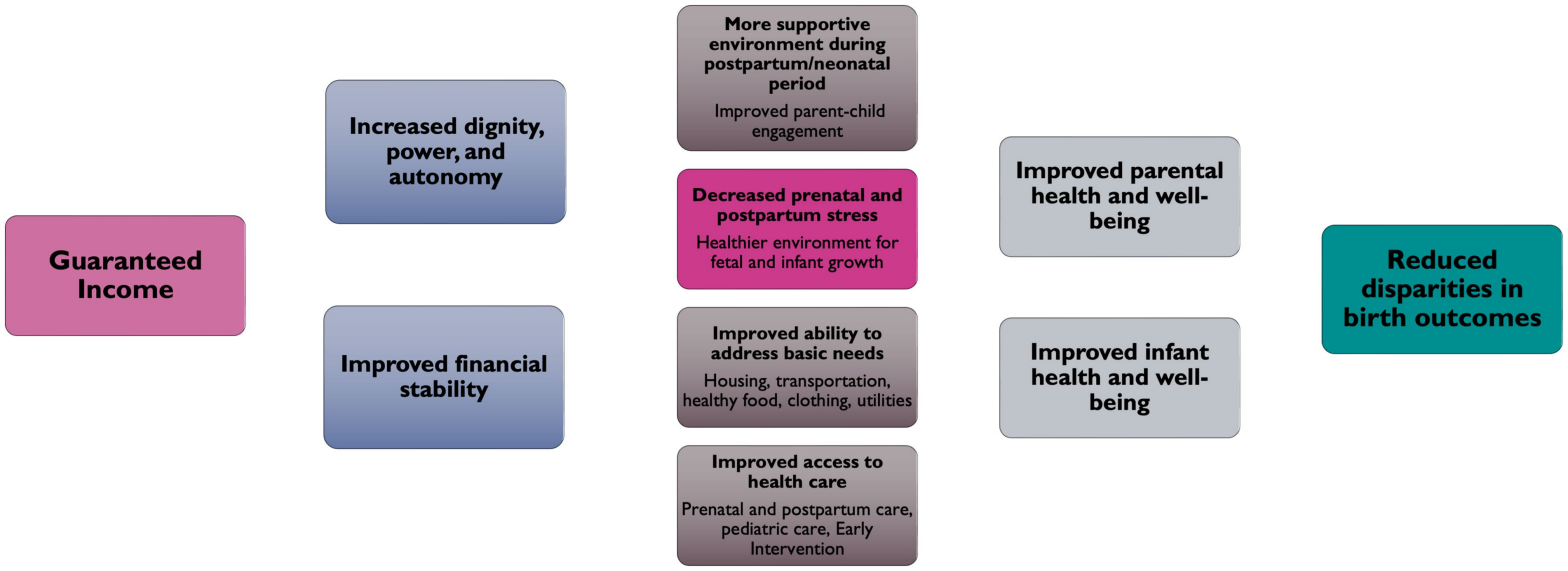
Pathways through which GI affects parent and infant health.

ReACH drafted several conceptual models of PJB’s theory of change, with the goal of informing both programmatic design and evaluation aims. The conceptual models were then presented to the HMH workgroup to ensure that the final conceptual model resonated with LEEs. As seen in Figure 1, the conceptual model illustrates the pathways through which providing cash to birthing people may influence intermediate outcomes, namely, by decreasing parental stress and increasing birthing people’s ability to meet their basic needs, improving their engagement with healthcare, and increasing attachment. It is posited that these changes will improve parental mental health and reduce infant prematurity in Philadelphia.

#### Community-led program design

Guided by the formative research and conceptual model, LEEs and other maternal and child health stakeholders of the HMH workgroup made key decisions to finalize the structure and amount of the GI, the geographic focus of the program, the protection of participants’ public benefits, and the provision of adjunctive services.

First, in alignment with other maternal and infant health programs, including perinatal services offered by ReACH, the program length was set to start in pregnancy and last until 12 months postpartum. To provide a benefit that was responsive to the needs of Philadelphia families, the amount of the PJB guaranteed income was set at $1,000 per month, informed by the Asset-Limited, Income-Constrained, Employed (ALICE) Household Stability Budget (20) that estimates $1,000 monthly housing costs in Philadelphia. To account for individuals with the lowest income as well as individuals who still struggle to meet basic needs, the income threshold was set at $100,000, informed by both ALICE and the Living Wage Calculator (21) that estimates an annual income of $100,000 is needed to support a household with two children in Philadelphia.

Second, given community feedback that Philadelphia is a city of neighborhoods, which do not align with zip code or census tract boundaries, a neighborhood analysis was conducted to identify neighborhoods in Philadelphia with the highest percentage of very low birth weight (<1,500 grams a proxy for prematurity) which are Cobbs Creek, Nicetown-Tioga, and Strawberry Mansion.

Third, to determine how to best protect recipients from the “benefits cliff,” in which a small increase in income can lead to a loss of public benefits, ReACH solicited information and input from other pilot programs through the Guaranteed Income Community of Practice^1^ and the City of Philadelphia’s collaborative on GI pilots. Together with other City of Philadelphia GI pilots, PJB sought and was granted state waivers that excluded PJB from eligibility calculations for most public benefits. To mitigate loss of other benefits relevant to the target population, such as childcare subsidy, PJB also contracted with a local agency to offer both group and individualized benefits counseling for all participants prior to enrollment into PJB. And finally, to augment the cash but support the unconditional, unrestricted nature of GI, perinatal and financial coaching services were added as voluntary supports that participants could opt into on an individual basis.

#### PJB steering committee and community-led program implementation

Approximately one and a half years prior to the launch of PJB, a dedicated PJB Steering Committee was created to further develop and finalize all aspects of PJB program design and implementation. The PJB Steering Committee is comprised of maternal and pediatric health practitioners, researchers focused on reproductive justice, leaders from community organizations, policy experts, and most importantly, LEEs from the HMH workgroup and/or each of the three target neighborhoods. Co-chaired by the PJB program manager and the LEE co-chair from HMH, the PJB Steering Committee has met monthly since its inception and uses CAN’s decision-making process to center LEE voices and votes.

Philly Joy Bank was finalized to provide $1,000 a month starting in the second trimester of pregnancy through 1 year postpartum for 250 pregnant people whose household incomes were less than $100,000 and who reside in the three target neighborhoods. By providing unconditional, unrestricted cash, PJB centers the dignity, power, and autonomy of participants to use the money as they see fit. Furthermore, to account for increased risks of fetal loss during the first trimester (22), yet maximize exposure to cash during pregnancy, the Steering Committee decided to restrict applicant eligibility to pregnant people in their second trimester of pregnancy.

Following the initial program design, the PJB Steering Committee embarked on a series of key implementation decisions. This included creation of a PJB Navigator position (to support applicants through the application process and to support participants in connecting with perinatal services), hiring and selection of the program manager, selection of disbursement, media, and storytelling vendors, and community-based outreach to recruit applicants for the program. The PJB Steering committee was not interested in selecting a disbursement vendor that tracked how recipients spent their cash as they felt that existing research had established that people who received unconditional cash spend their money to meet their family’s needs. The LEE co-chair was involved in all interviews and hiring processes, and the same consensus-based decision-making processes were used to interview and select final vendors for collaboration and support of PJB.

### Part 2. Development of the PJB evaluation

Consistent with the broader organizational structure that guides PJB and consistent with the research team’s own approach to evaluation, the PJB evaluation has been developed using a participatory process (23, 24). This process began with the selection of the research team (Drexel University) as the evaluation partner and continued through the development of the evaluation itself and in data collection and analysis.

The PJB Steering Committee identified what they hoped to learn about the impacts of PJB and subsequently issued a request for evaluation proposals (RFP). The RFP indicated that (1) the evaluation should not be conducted as randomized controlled trial so that PJB applicants did not have to join a research study to be eligible for cash; and (2) the evaluation should include narrative-based methods (like photovoice) to elevate the voices of individuals receiving cash. Before reviewing proposals, community members on the PJB Steering Committee took part in a “Research 101” training. This training prepared them to review proposals by providing an overview of different study designs and research methods commonly used to evaluate health impacts. The training included a discussion of the pros and cons of potential study designs. After the training, members of the PJB Steering Committee felt that a quasi-experimental mixed methods study design was the approach most aligned with the underlying values of the community-led evaluation: it was rigorous, it centered the voices of lived experience experts, and it protected the rights of PJB applicants not to engage in research.

The PJB Steering Committee selected the top three research teams to pitch their proposed evaluation plan at a subsequent meeting. Following standard community-centered processes, the PJB Steering Committee discussed the pros and potential ethical concerns of each evaluation plan, and the strengths and weaknesses of the potential evaluation teams. At the conclusion of the discussion and prior to voting, the PJB Steering Committee provided space for LEEs to voice any additional concerns they might have about the proposed study designs and research teams.

The research team selected by the PJB Steering Committee is comprised of a group of individuals with relevant research experience and lived experience. Collectively, most of the team’s research focuses on promoting reproductive justice, here in Philadelphia and across the globe. The research team has complementary expertise and significant history in community-based participatory research methods (CBPR), including collaborative program design, implementation, and evaluation with its community partners.

#### Community advisory board

After selecting the Drexel University research team as the evaluation partner, the PJB Steering Committee formed a Community Advisory Board (CAB) composed of LEEs and PJB backbone staff from ReACH (e.g., PJB program manager and navigator, Philly CAN Manager, Perinatal Surveillance Epidemiologist, Child Health Policy Advisor). Since October 2023, the CAB has met with the evaluation research team to co-design the PJB evaluation. The research team uses CBPR as an established approach suited for conducting research in partnership with historically marginalized groups for evaluation (23, 24). CBPR prioritizes partnership between community members, organizational representatives, and academic researchers, all of whom contribute their expertise and share responsibility and ownership at each stage of the evaluation process (23, 24). Thus, following the same approach used by the PJB Steering Committee, key features of the evaluation design and related research processes were presented to the CAB to elicit and integrate their feedback.

#### Community-selected study design

Congruent with the community-driven design of PJB, the evaluation of the impact of PJB is grounded in the principles of CBPR and is co-designed by the community. The design involves (a) quasi experimental methods to enroll participants into an intervention group (*n* = 150 selected by lottery to receive cash + care) and a comparison group (*n* = 150 who applied for but were not selected by lottery and thus will receive care only (financial counseling, lactation, doula, and home visiting)) and (b) longitudinal participatory qualitative research methods with a subset of participants from the cash + care group (*n* = 25). The Steering Committee emphasized their preference for narrative-based research methods to elevate the voice of cash transfer recipients, which led to the selection of qualitative participatory arts-based research methods (e.g., Photovoice, cellphilms) as part of the mixed methods design. The purpose of the study is to assess the feasibility and acceptability of PJB (Aim 1); to examine *whether* and *how* a guaranteed income affects ability to meet basic needs and parental stress (Aim 2); and to explore the preliminary impact of PJB on parental mental health and prematurity (low birth weight and preterm birth) (Aim 3). Descriptive statistics, mixed effects regression analyses, and participatory qualitative analysis approaches will be used to achieve study aims.

#### Community involvement in instrument development

First, the research team designed the survey instruments with the CAB. For example, at the first meeting regarding the development of the baseline survey, all potential domains to be covered in the baseline survey were discussed with the CAB, including those relevant to key outcomes (e.g., mental health), those identified by the CAB that might also be influenced by receipt of GI (e.g., use of a doula), and those that the CAB felt were not acceptable (e.g., substance use and extensive questions about how the money was spent). The research team then consulted the literature and identified potential validated measures for selected domains with a preference for measures used in other GI programs and/or measures validated with Black pregnant populations. In subsequent CAB meetings, the research team presented up to three measures for each domain, and the CAB discussed and voted on their preferred measure. In some cases (e.g., housing instability), the CAB requested alternative measures. The research team then identified different measures and presented them again to the CAB for approval. In other cases (e.g., intimate partner violence), the CAB felt that the three proposed measures did not speak to their lived experience. Thus, for these domains, the research team co-developed new items with the CAB. After the completion of each survey, the CAB members pilot the survey and provide additional feedback on the process. This iterative feedback process results in surveys that are tailored to the community and are responsive to the lived experience of Black birthing people in Philadelphia.

#### Community involvement in planning the participatory research sessions

Second, the research team designed the structure of the participatory research sessions with the CAB. Prior to the start of the participatory research sessions, we held an in-person training session with all members of the CAB to describe how the method worked. After the overview, members of the CAB participated in a mock Photovoice session. The mock session was facilitated by two LEEs who were selected to co-lead the participatory sessions. After learning about the method, the CAB helped guide decision making about the timing of the sessions (weekend vs. evening), preferred location for the sessions, and the total number of sessions that would be offered for each phase of data collection.

#### Community involvement in recruitment and retention processes

Third, recruitment and retention processes were designed with feedback from the CAB. For example, CAB members helped to decide when in the application process the research team could contact individuals to engage them in the evaluation study. CAB members also provided feedback on scripts to use when contacting potential (or current) research participants.

#### Community involvement in data collection and analysis

Fourth, the research team and CAB include community members in all aspects of data collection and analysis. For example, surveyors are women who live in the communities we serve, members of the CAB help to facilitate the participatory sessions, and the individuals in the participatory sessions are co-researchers. This process helps to ensure that the voices of the community members are centered in the research process, and especially cash transfer recipients in data collection and analyses. This process will also elevate the lived experience of cash transfer recipients in dissemination and advocacy.

### Part 3. Implementation of PJB

#### Study setting

PJB takes place in the city of Philadelphia, which – with a poverty rate of 21.7% and even higher poverty rates among Black families (25%) – is “America’s poorest big city” (25). More specifically, to apply for PJB, residents must live in one of the following three low-income and predominantly Black neighborhoods in Philadelphia: Cobbs Creek, Strawberry Mansion, and Nicetown-Tioga. These neighborhoods are characterized by high levels of poverty (median family income ranges from $35,671–$41,764), high levels of inadequate prenatal care (36.6–37.3%), poor birth outcomes (14.1–14.8% of infants are low birthweight), and poor educational performance (40.6–47.5% of children K-12 are reading proficient) (26).

#### Application to PJB

**Figure 2:**
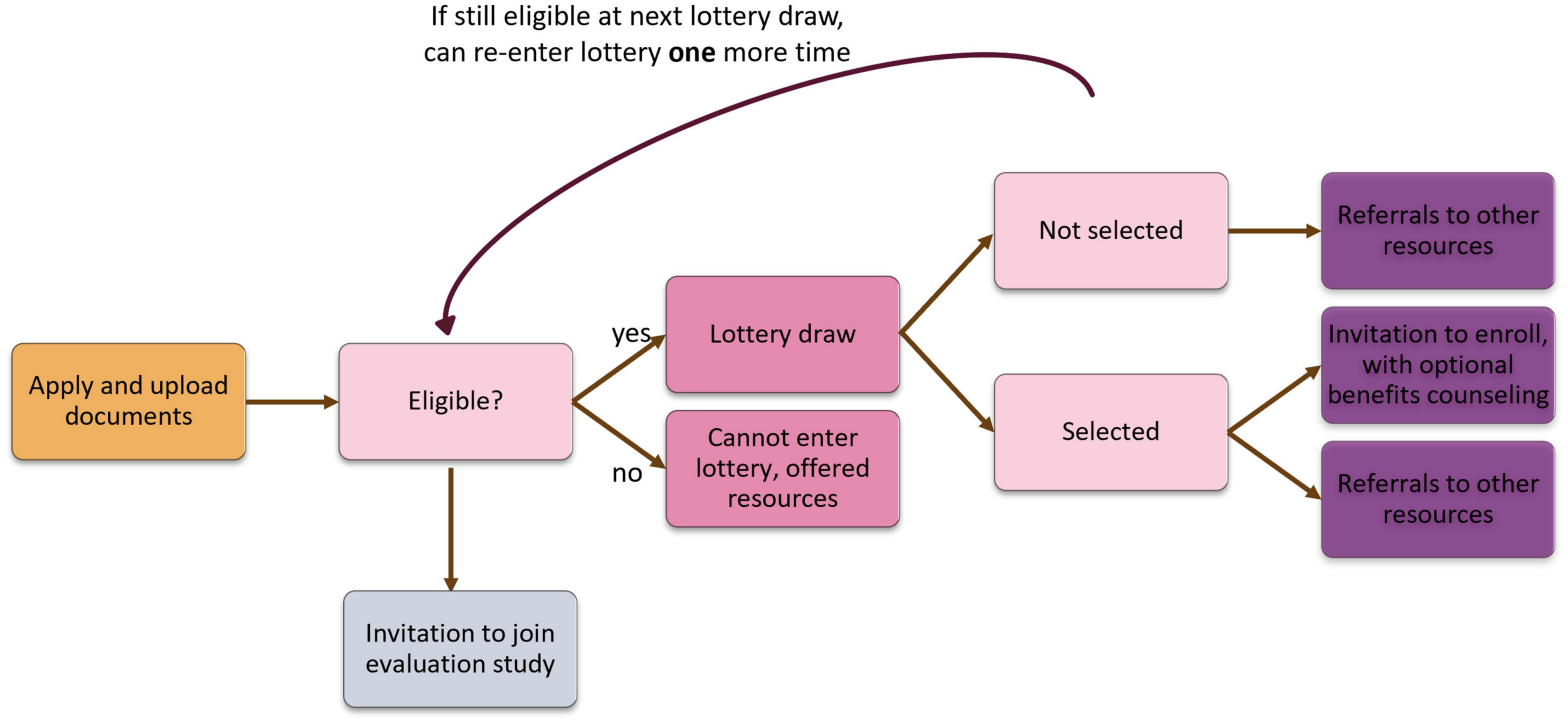
Process of selecting applicants for program and evaluation.

Applicants for PJB are recruited using a three-tiered recruitment process, which was developed by the PJB Steering Committee: (1) referral and advertisement at healthcare providers in each neighborhood; (2) referral and advertisement at neighborhood community sites (social services offices, libraries, etc.); and (3) via social media, including the Philly Joy Bank website^2^. Individuals interested in applying for the PJB lottery do so via the PJB website, where they complete their application and receive assistance with submitting their application, if needed. Individuals can apply for the PJB lottery if they are: (a) 18 years or older; (b) between 12 and 24 weeks pregnant; (c) self-attest to household income of ≤100 K per year; and (d) live in one of the three target neighborhoods. The PJB program manager and navigator confirm each applicant’s eligibility for PJB before applicants are recruited to participate in the evaluation research study or entered into the lottery. See Figure 2 for a visual of the timeline of PJB application, lottery selection, and research study enrollment.

#### Lottery selection process

All eligible applicants are subsequently entered into a lottery to enroll in PJB (cash + care). The PJB Steering Committee voted on selection via lottery for three reasons. Specifically, they felt that (1) interest in PJB would exceed availability; (2) random selection would reduce bias in selection of participants; (3) it was difficult to determine who is most “deserving” of the money given high needs across most of the eligible population. Everyone who is selected into PJB via the lottery receives “cash + care,” a monthly cash disbursement and optional financial counseling and care services (doula, lactation, home visiting). Individuals who are not selected are eligible to receive “care” i.e., optional financial counseling and care services. Eligible applicants for PJB are selected at a probability that was determined by the PJB Steering Committee stratified by neighborhood. Random selection takes place every 2 weeks using the current eligible applicant pool for the PJB until 250 participants are selected by lottery to receive cash + care. An applicant may be entered into a second lottery draw if they are still eligible (by gestational age) at the time of the second lottery. At each lottery draw, the PJB implementation team utilizes a neighborhood-stratified simple random sample method to select eligible applicants from each neighborhood. Individuals who are selected for PJB are notified via automated text and email, then a phone call by the PJB Navigator and given 2 weeks to enroll in PJB. Enrollment of 250 participants into PJB began in June 2024 and was completed in March 2025.

### Part 4. Evaluation of PJB

#### Research study enrollment and retention

Prior to lottery selection, all individuals who are eligible for the lottery are also eligible (but not required) to participate in the evaluation research study. Individuals learn about the opportunity to join the study from community researchers (CR) immediately after the PJB team confirms their eligibility and prior to lottery selection. The CRs emphasize to applicants that study participation is voluntary -- lottery selection is not contingent upon participation in the study. Individuals who are interested in participating in the study provide informed consent via phone and either complete the baseline survey the same day or schedule a time for baseline survey completion within the next week. At the time of the baseline survey, approximate dates for the follow-up surveys are scheduled based on the participant’s estimated date of delivery (EDD). At enrollment into the study, participants are asked to share their phone number and their address so that we may remind them of future appointments.

Following approaches the research team has used in other studies, CRs check in with participants via phone, text message or email at a minimum of four time points to maximize retention. First, CRs do a phone check-in with participants 1 month prior to delivery and also send out a short e-newsletter around the EDD to keep participants passively engaged and foster a sense of community and commitment. Second, CRs do a text or call check-in with participants 1 month after estimated delivery to ascertain actual delivery date. CRs repeat the phone check-in approximately 1 month prior to all postpartum visits to confirm or (re)schedule the follow-up survey. Participants who miss their scheduled appointment are contacted within one business day. At least four attempts are made to contact the participant by phone, text or email, using multiple contact times to increase success (am/pm/weekend).

#### Quantitative data collection

**Figure 3:**
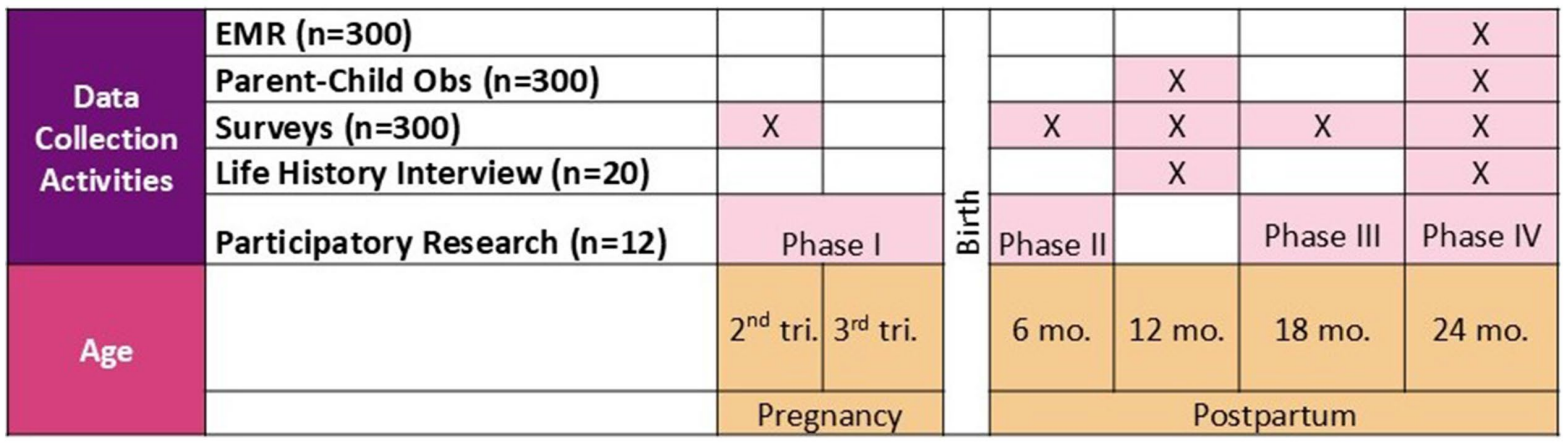
Philly Joy Bank evaluation time points.

Quantitative data collection takes place at enrollment (12–24 weeks gestation) and at 6 and 12 months postpartum (Figure 3). Participants in both groups (i.e., cash + care and care only) complete the baseline survey prior to receiving the cash disbursement, financial counseling, or care services. Survey data is collected through REDCap, a secure, web-based application that complies with HIPAA regulations. All data collection procedures follow approaches used in other local studies. Each survey is administered via an interviewer over the phone. Each question is read to the participant; responses are recorded on a tablet with a secure internet connection. All data management standards reflect the goal of reproducible research. To reduce data entry errors and omissions, we take advantage of REDCap’s built-in quality control functions. Data integrity is aided by the system’s automated logging of all input and edits. After each survey is complete, CRs send the participant a gift card (electronically or by text) and a community resource guide. Participants receive $50 for completion of the baseline survey, and the amount is increased by $25 for each subsequent survey.

#### Quantitative measures

Outcomes will come from multiple data sources. We will draw on PJB routine monitoring data to assess feasibility. Self-report outcomes (including reports on acceptability and satisfaction) will come from surveys. Finally, for engagement in prenatal care and birth outcomes, with consent from participants and permission from the Pennsylvania Department of Health, Bureau of Health Statistics and Registries, we will obtain individually matched birth records of study participants.

#### Feasibility and acceptability

Reach will be assessed using monitoring data collected by PDPH, and will include number of applications, number of eligible applicants, number invited to enroll, number enrolled in PJB, and number referred for care services. Dose received will be tracked by examining how many payments each participant was eligible to receive and how many they received over the course of the program. Retention of participants in the evaluation study will be reported for each evaluation time point. Acceptability will be assessed using survey items at the 12-month endline survey and participatory data.

#### Outcomes

Low birth weight will be extracted from the birth record and defined as an absolute weight of <2,500 g regardless of gestational age (27). Preterm birth will be extracted from the birth record and defined as birth prior to 37 weeks gestation (27).

Depression will be assessed using the Edinburgh Postnatal Depression Scale (EPDS), a measure validated for use during and up to 24 months after pregnancy (28–31). The scale consists of 10 items scored on a 4-point Likert scale which ask about common depressive symptoms experienced in the prior week. We will characterize this primary outcome using a continuous score because subclinical symptoms can impact functioning (32, 33). Additionally, we will characterize probable depression (yes vs. no) using a cut-off score of ≥11 (34). Anxiety will be assessed using the Generalized Anxiety Disorder scale-7 (GAD-7) score (continuous) which is a reliable and validated measure for use during and after pregnancy. The GAD-7 consists of 7 items scored on a 4-point Likert scale which ask about common anxiety symptoms in the prior 2 weeks (range 0–21).

Stress will be measured through two scales. First, perceived stress will be measured with the ten-item Perceived Stress Scale (PSS) (35). Scores range from 0–40 and will be categorized into three groups as low stress (0–13), moderate stress (14–26), and high stress (27–40). Second, parental stress will be measured using three modified subscales of The Parental Cognitions and Conduct Toward the Infant Scale (PACOTIS), including: parental self-efficacy (six items), perceived parental impact (five items), and parental overprotection (four items) (36). Items are scored from 0 to 10 with higher scores indicating higher levels of self-efficacy, parental impact, and overprotection. Mean scores for each subscale will be used for analysis. Ability to meet basic needs will be measured through three domains: financial, food, and housing insecurity. Financial insecurity will be measured using the 10-item Consumer Financial Protection Bureau Financial Wellbeing Scale (37). Continuous scores ranging from 0 to 20 will be categorized by quartiles for analysis (38). The validated, two question Hunger Vital Sign screener (39) will be used to measure households at risk of food insecurity, and housing insecurity will be assessed using up to 16 items on history of homelessness, history of eviction, and falling behind on rent and utilities. Engagement in health care will be captured using prenatal care attendance data from the birth certificates. Specifically, adequacy of prenatal care (APCNU) is assessed using the Kotelchuck index by creating a ratio of observed prenatal care visits to expected prenatal care visits, relative to the American College of Gynecology recommended standard of care, classified as inadequate, intermediate, adequate or adequate plus (40). Parent–child attachment will be assessed using the Maternal Postnatal Attachment measure, a validated continuous scale (41). The scale consists of 19 items which ask the birthing parent to reflect on their thoughts and feelings toward their baby (42).

#### Quantitative data analysis

##### Preliminary and descriptive analyses

All data will first be assessed for missingness and out-of-range values with basic statistical procedures such as univariate statistics (means, standard deviations, ranges, frequencies, proportions, percentiles) and graphs such as histograms, box and whisker plots, scatter plots and Q-Q plots. In addition, plots may be produced of individual and average trajectories of all repeated measures over time according to assigned treatment. All questions of data quality and integrity will be investigated before any statistical modeling, as complete and accurate data are essential for unbiased estimates and confidence intervals. Baseline and demographic characteristics will be summarized overall for all participants and by intervention group. Univariate summary statistics will be provided for continuous variables and frequencies for categorical variables. Graphical methods mentioned above will be used to examine distributions and identify potential influential points. Assumptions of all statistical models for the aims will be assessed; data transformations for variables may be considered in analyses if required to meet assumptions of the statistical tests/models. Missing data will be assessed, and sensitivity analyses will be considered. Scale data where participants answered at least 80% of the items will be imputed using person-mean substitution. In instances where a participant omits more than 80% of the items from a specific scale, responses from that scale will be excluded from analysis.

###### Aim 1

To assess the feasibility and acceptability of PJB, we will use routinely collected data on study engagement among potential participants and 12-month endline survey data among PJB participants. Quantitative data analysis: Feasibility: We will assess the number and proportion of participants reached by PJB by creating quarterly reports from the routine data. We will compile absolute numbers for the following: (1) # who indicate interest; (2) # invited to enroll; (3) # who enroll; (4) # who are referred for care services. Acceptability: We will describe perceived acceptability quantitatively using 11 items on spending and decision-making control and ten items about the timing of GI payments from the endline survey. Acceptability of each care service will be evaluated using six items from the endline survey and acceptability of PJB and its evaluation will be assessed using three survey items. Qualitative data analysis: We will also examine perceived acceptability in the participatory session and will analyze data following procedures in Aim 2.

###### Aim 2

Examine *whether* and *how* a guaranteed income affects parental stress, ability to meet basic needs, engagement in healthcare and bandwidth for parenting among recipients of the cash + care. Quantitative data analysis: we will use survey data to examine *whether* receiving cash + care affects outcomes using two steps. First, we will describe baseline demographic characteristics among those who receive cash + care. Second, we will examine differences in parental stress, basic needs, engagement in care and bandwidth for parenting over follow-up (baseline, 6 months postpartum, and 12 months postpartum surveys). We will use mixed effects regression models with a fixed effect for time (categorical) and random participant-level intercepts. We will examine the sensitivity of our findings to the inclusion of key potential confounders in the model, including SES and pregnancy-related variables (e.g., gravidity, parity, maternal health conditions). Using these models, we will estimate and report the average difference in outcome between groups over follow-up with 95% confidence intervals. We will also characterize outcome patterns over time by presenting estimated mean outcomes at each time point individually.

###### Aim 3

Explore the preliminary impact of PJB on parental mental health and prematurity. The purpose of the PJB is to reduce disparities in birth outcomes. Given the limited numbers of participants in this pilot program and evaluation study, however, we are underpowered to examine such disparities. Consequently, birth outcomes are included as exploratory analysis for potential signals of GI impact. Another important outcome for the PJB evaluation study is perinatal depression, which we do have sufficient statistical power to examine. Perinatal depression is also associated with adverse birth outcomes and can be a marker for perinatal health.

To investigate post-baseline differences in continuous EPDS depression score in the cash + care and care groups, our primary analyses will use mixed effects regression models with fixed effects for time (baseline, 6 months postpartum and 12 months postpartum), group, and the interaction between time and group and random participant-level intercepts. Using these models, we will estimate and report the average difference in depression score between groups over follow-up with 95% confidence intervals. We will also characterize outcome patterns over time by presenting estimated mean outcomes at each time point individually. We will use the same approach to examine post-intervention differences anxiety score. In secondary analyses, we will examine differences in infant birthweight and preterm birth in the cash + care vs. care groups using logistic regression. Birthweight and gestational age at birth will be determined using data from birth records and classified as low birthweight <2,500 grams (y/n) and preterm birth <37 weeks (y/n). We will examine the sensitivity of our findings to the inclusion of key potential confounders in our models, including SES and pregnancy-related variables (e.g., gravidity, parity, maternal health conditions).

#### Power and sample size

This study is powered on detecting a difference in EPDS depression score between the cash + care vs. care groups during the 6- to 12-month postpartum period (Aim 3). Preliminary data from eligible participants suggest a mean Edinburgh Postnatal Depression Scale (EPDS) score of 10.45. Table 1 presents the estimated minimum detectable differences in depression scores between the two groups given a total of 281 participants with 164 in the cash + care group and 117 in the care-only group. We assumed two observations per participant (6- and 12-months postpartum) after initiation of the cash + care or care only interventions, a standard deviation of 6.24 (based on preliminary data), a significance level (two-sided alpha) of 0.05, and a within-participant correlation ranging from 0.3 to 0.5. For example, we achieve 80% power to detect a 1.8 point mean difference in EPDS score between the care-only group and the cash + care group, given a within-participant correlation of 0.5. A clinically meaningful difference on the EPDS scale is defined as a change of 4 points for improvement (43). Given our current sample size, we will have sufficient power to detect these clinically significant differences. Even with a projected 25% dropout rate for both groups (e.g., 123 in the cash + care group and 88 in the care-only group), we will still be able to detect a minimum difference of 2.1. All power calculations were carried out using PASS (44).

**Table 1:** Estimated minimum detectable differences in depression scores.

Within-participant correlation	Mean difference
0.5	1.8
0.4	1.8
0.3	1.7

#### Overview of participatory qualitative research methods

Longitudinal participatory arts-based narrative methods, hereafter participatory research, will be used to identify potential mechanisms affecting mental health and infant health from the perspectives of PJB recipients (45, 46). Findings will also be used to contextualize the quantitative findings. Per the tenets of participatory research, involving community members who are the focus of research throughout the entire research process enhances the identification of the research topic, the research process, and the impact of findings (47). Thus, the participatory research component has been co-designed with the members of the CAB, and participatory methods will be applied to the data collection, analysis, and dissemination of findings. The reflexive longitudinal participatory design prioritizes opportunities for (a) participant feedback in all aspects of the research process, (b) building ongoing relationships with participants, and (c) ongoing analysis with participants throughout data collection (48). Further, relationship-building between participants and group cohesion are inherent to the proposed methods. Relevant to the study’s focus on structural racism and financial insecurity among Black birthing people, participatory research methods have been used in such areas as structural and neighborhood level factors affecting Black mothers’ physical and mental health (49–51).

#### Participatory data collection

The data collection will occur in two phases: during pregnancy (phase I), and 6 months postpartum (phase II). Phase I includes six 2-h weekly in-person group sessions. Phase II includes 2 three-hour sessions. Because cohesion will have been established during Phase I, and because participants will have an infant/child, Phase II will be shortened to two 3-h sessions to maximize retention. Sessions will occur at a site chosen in collaboration with participants, with meals, transportation and childcare included and will likely occur during weekends to maximize participation. Participants will be compensated for their time ($25/h). All sessions will be recorded with consent. Sessions will be led by the LEEs from the CAB, with guidance from the co-principal investigator (Valdez). We will use the methodological framework of *critical narrative intervention* in this study and envision using a blend of two participatory arts-based research processes – Photovoice and Cellphilms (52–54). In *Photovoice*, participants use photographs and other visual representations of their daily lives to explain their lived experiences (54). Photovoice has been used with other racialized birthing populations (55) and provides an opportunity for participants to share their opinions about and analyze health issues that may be silenced by societal, clinical, community, and family structures. *Cellphilms* are brief films made on cellular devices by participants in response to prompts that tap into the individual’s already well-developed expertise in analyzing their everyday lives (52). In Photovoice and Cellphilms, participants define their own photo prompts, conduct data collection (photography, filming), and engage in group thematic analysis based on their productions (photos, short films). Both methods use Storytelling, shown to preserve a collective memory and share historical knowledge (55–57). We will use Photovoice/Cellphilm methods to obtain nuanced pictures, videos, and corresponding narratives of participant’s lives during and after receipt of PJB. Subsidies will be made available to participants to account for any lack of access to data coverage and/or cell phone. All narrative and photo data will be stored in a secure location and all identifying information will be removed.

Phase I (during pregnancy) will include 6 sessions as is typical in participatory research using Photovoice/Cellphilms (52, 54). As seen in Table 2, Session 1 will involve identity and community building to establish rapport between participants and the research team (ESV, LEEs), as well as familiarizing the group with the ethical issues involved in photographing/filming others, the potential risks to participants in taking photos/videos and how to minimize these risks, the practice of giving photos back to the participants, and a review of photography techniques using a cell phone camera (53). Participants will receive guidance on how to generate photos/Cellphilms in response to the prompts to explore if/how GI influences (1) perceptions of financial security; (2) stress; and (3) ability to meet basic needs. At the end of Session 1, participants will collectively select one prompt as the focus of the next week’s assignment. Participants will take photos/videos of the prompt while going about their weekly activities and will use the SHOWED method to describe each photo/Cellphilm (52–54). The SHOWED method consists of five questions that lead participants through the photos/Cellphilms: (1) What do you see here?; (2) What is really happening here?; (3) How does this relate to your life?; (4) Why does this situation, concern, or strength exist?; and (5) What can we do about it? At each subsequent session (Sessions 2–4), group discussion, or “debriefs” will provide insight into the influence of GI on the aforementioned prompts. In each session, participants will also explore the mechanistic relationships between prompts (e.g., perceptions of financial security, stress, ability to meet basic needs) with (a) mental health and (b) their relationship to their child. Session 5 will involve group thematic analysis and Session 6 will be a group celebration.

**Table 2:** Participatory research sessions, by phase.

	Session 1	Session 2	Session 3	Session 4	Session 5	Session 6
Phase I	- Introduce activities - Assign prompt #1	- Debrief prompt #1 - Connect prompt to outcomes - Assign prompt #2	- Debrief prompt #2 - Connect prompt to outcomes - Assign prompt #3	- Debrief prompt #3 - Connect prompt to outcomes	- Group thematic analysis	- Celebration (completion of Phase I)

	Session 1	Session 2
Phase II	- Reconnect - Debrief prompts #1–3 (assigned ahead of time) - Connect prompts to outcomes	- Group thematic analysis - Celebration (completion of participatory study)

In Phase II (6 months postpartum), we will ask participants to reflect on how their lives have changed during PJB (Phase II) using Photovoice/Cellphilm methods, especially in relation to the aforementioned prompts. For these sessions, participants will be asked to revisit prompts in advance and bring photos/Cellphilms to the debrief. Participants may share how the cash was used in the photovoice sessions in response to prompts. In Session 1, we will debrief using the new material. As part of the debrief, we will incorporate visual and narrative data from prior phases, as connecting data across time allows us to understand change more than any single time point (48). Group discussion will also include connecting prompts to outcomes. Session 2 will involve group thematic analysis.

#### Qualitative data analysis

We will use the Photovoice and Cellphilm qualitative data to assess *how* receiving cash + care affects outcomes using three steps. Qualitative data analysis will involve an emic (i.e., insider’s perspective) analysis of the Photovoice data described above (53, 58). Participants will engage in individual reflection and group discussion of photographs/Cellphilms. Each participant will select three photos and/or one Cellphilm to present from their photo/Cellphilm expedition for each debrief session along with titles and SHOWED narratives to share with the larger group. Thus, a maximum of 75 photos/ 25 Cellphilms will be selected and discussed by the 25 participants using the SHOWED narrative approach across the 8 sessions. As part of the group thematic analysis, the evaluation team and participants will identify emergent themes and corresponding definitions arising from the photos/Cellphilms, narratives, and discussions at both phases (prenatal and postpartum). Second, the evaluation team will conduct an etic (i.e., outsider’s perspective) analysis of data (58), wherein we will code the photos, Cellphilms, SHOWED narratives, and transcripts of recorded group discussions for salient themes relating to the prompts/research questions. Third, upon completion of both emic/participant and etic/academic researcher analysis, the evaluation team will conduct a triangulation of themes that emerged in the coding of Photovoice and Cellphilm data itself, and the coding of participants’ analysis and discussion of their Photovoice/Cellphilm data. Participants will contribute to analysis during etic processing and debriefing, including Photovoice/Cellphilm activities during the sessions. Iterative etic data analysis will occur after sessions by the evaluation team. The combination of group discussion and Photovoice data integrates a critical approach to participatory analysis with traditional qualitative research practice (53).

## Discussion

PJB is a GI program in which pregnant individuals residing in three low-income, predominantly Black neighborhoods will receive unconditional cash payments during pregnancy and the first 12 months postpartum. The overarching goal of this community-led mixed-methods evaluation study is to determine the effects of PJB on birthing parents’ mental health and their infants’ health during pregnancy and through the first year postpartum. This is a critical and under-examined issue, considering that Black birthing people and their children experience high levels of economic insecurity and significant health disparities during this formative period in the life course.

GI programs continue to increase in the U.S., and there are several indications that providing GI in the perinatal period may be replicable and scalable. For example, the Bridge Project, which started in New York City, has been replicated in 8 other states. In addition, 2 other GI programs have been scaled up after successful pilots. Specifically, Rx Kids first started in Flint, Michigan, and is now offered in dozens of new communities across the state (59), and the Abundant Birth Project (60) first started in San Francisco, California, and is now offered in 5 additional counties. In addition, the existence of several other national and/or state cash transfers (e.g., expanded child tax credit in 2021, Alaska Permanent Dividend Fund, Baby Bonds) that are not conditioned on earnings also highlight both feasibility and political will for these types of programs across political contexts.

Despite increased interest in unconditional cash transfers (like GI) in the U.S., evidence regarding the impacts of these programs on infant and parent health in the U.S. is limited. A recent study of RxKids, which gives pregnant women $1,500 during pregnancy and $500 a month for a year postpartum, indicated that the cash transfer had a positive impact on maternal mental health (61). On the other hand, a study of Baby’s First Years, which takes place in NYC, New Orleans, Omaha, and the Twin Cities, and provides families with $333 per month for the first 76 months of a child’s life, found no differences in psychological distress between recipients and non-recipients of the GI (62). A different empirical study not specific to the perinatal period found that recurring unconditional cash transfers in the U. S. during the COVID-19 pandemic to families living in poverty improved parental mental health, particularly for Black Americans (63). And finally, simulated models examining multiple safety net programs in the U. S. suggest that a more generous safety net is protective of maternal mental health. For example, a $1,000 increase in cash and food benefits reduces severe psychological distress among single mothers by nearly 10 percent (64).

The evidence regarding the impacts of GI on birth outcomes and infant health in North America is favorable, but sparse. Specifically, in Manitoba, Canada, a GI program which provided $81/month to low-income First Nations women during pregnancy positively influenced birth outcomes (65). A GI pilot in Massachusetts with mothers who had infants in the neonatal intensive care unit (NICU) found that mothers randomized to receive $200 per week were more likely to visit their infants in the NICU, provide skin-to-skin care, and provide breastmilk than mothers who did not receive the GI (66). Finally, BFY has reported limited impact on infant health (i.e., no differences between groups in mother’s reports of ER/urgent care visits, well-child visits) (67) and largely null findings on children’s language or socioemotional development (68, 69). However, given the limited evaluation of U.S. programs, as well as the variability in existing GI program components (e.g., the timing of when GI is provided, the amount and duration of GI that is provided, and eligibility criteria for recipients), further research on impacts on maternal health, infant health and child development is warranted.

Further, there is limited understanding of the durability of GI programs on health outcomes in general (i.e., do the protective effects of GI occur during or after GI, and are they sustained after the cash transfers have ended?). Relatedly, no studies examine whether GI’s protective effects are strengthened for subgroups facing heightened financial insecurity (e.g., those facing costly infant hospitalization in a NICU, single parents, parents with the lowest SES, or parents with more than one child). Finally, understanding how GI impacts health for the most marginalized groups requires centering their voices. Participatory qualitative methods are ideal methods for illuminating the complex structural and institutional contexts (70) that shape whether and how Black birthing people can benefit from GI. This study will address these gaps.

### Limitations

There are several potential limitations to the PJB evaluation study. First, there may be challenges retaining the cohort over time (particularly participants in the care only arm). To mitigate this risk, CRs will engage in ongoing contact with participants throughout the study to maximize retention over time. Moreover, the sample is powered to detect a difference in depression outcomes even after accounting for potential attrition of up to 25%. Second, some of the parent–child relationship measures are self-report. Observational measures (i.e., the Strange Situation task, the Attachment Q-Set) are typically considered the “gold standard” for the assessment of attachment. However, self-report measures were intentionally selected because observational measures are time- and resource- intensive (71), are considered unethical by some, and do not consider the subjective experience of the parent (72). In addition, measures were selected to assess a range of parenting domains to gain a holistic perspective on the parent–child relationship.

### Strengths

Despite these potential limitations, the PJB evaluation study has several strengths. First, the focus on low-income and predominantly Black families during and after pregnancy is unique. Many GI pilots during the perinatal period provide cash in the postpartum period only and focus predominantly on the health impacts of GI for low-income children (65, 73). Research that centers parents’ lived experiences is vital to understanding how GI may impact parent and child health over time. Conducting this research in Philadelphia, where Black birthing people face especially high poverty rates, offers unique opportunities to strengthen insight into GI’s implications for health equity and inform local interventions. Second, the use of participatory arts-based narrative approaches will facilitate understanding of the pathways through which GI impacts health from the perspective of GI recipients (45, 46). The participatory methodology will identify potential mechanisms and important outcomes from the perspectives of those that receive GI, clarifying the understanding of why and how GI works and providing insights for future program implementation.

Finally, the study leverages an established, long-standing community partnership to address financial insecurity surrounding pregnancy and advance health equity in Philadelphia. PJB was generated through a community-driven strategic planning process, and all program details are decided by the PJB Steering Committee, half of whom are community members with lived experience as Black birthing people. It is one of the first of its kind to be conceptualized, designed, and implemented by the community, using an evaluation approach that is co-designed by the community and responsive to the lived experience of Black birthing people. Evaluating impacts of PJB – and co-disseminating the results alongside community partners – may inform future social policy, by highlighting the potential of programs (like GI) that support parents’ dignity and agency to raise thriving children. Such efforts are critical, given that traditional social policies do not always reach low-income families and communities of color.

## Conclusion

The proposed community-led mixed methods study will occur in a city where more than 25% of Black families live in poverty, where nearly 20% of Black parents report symptoms of depression after childbirth, and where over a quarter of infants have been hospitalized before their first birthday. Findings will contribute to a comprehensive understanding of how GI affects parent and infant health in three low-income and predominantly Black neighborhoods in Philadelphia, where historical and systematic restriction of access to vital resources has produced persistent health inequities. These contributions help inform emerging evidence that GI is a promising upstream approach to addressing persistent health inequities in the U.S.

## Footnotes

1
https://gicp.info/

2
https://www.phillyjoybank.org/

## References

[1] Martin J Hamilton B Osterman J. Births in the United States, 2020. NCHS Data Brief. (2021). 418, 1–8. doi: 10.15620 34582330

[2] Assibey-Mensah V Farley T Kallem S Sabol T Washington R. Growing up Philly: the health and well-being of Philadelphia’s children. Philadelphia, PA: Department of Public Health (2020).

[3] National Center for Health Statistics. Final natality data [internet]. Published online January 17 2025. Available online at: www.marchofdimes.org/peristats (Accessed August 20, 2025).

[4] Ely DM Driscoll AK. Infant mortality in the United States, 2022: Data from the period linked birth/infant death file. (2024). CDC Stacks, National Center for Health Statistics. Available online at: https://stacks.cdc.gov/view/cdc/231880 39680705

[5] Huynh MP Nyame-Mireku A Patel F. Depression among Pregnant & Postpartum Philadelphians. Philadelphia, PA: Philadelphia Department of Health.

[6] Bauman BL Ko JY Cox S D’angelo DV Warner L Folger S . Vital signs: postpartum depressive symptoms and provider discussions about perinatal depression—United States, 2018. MMWR Morb Mortal Wkly Rep. (2020) 69:575. doi: 10.15585/mmwr.mm6919a2 32407302 PMC7238954

[7] Mehta A Hoffman R Tew S Huynh M. Improving outcomes: Maternal mortality in Philadelphia. Philadelphia, PA: Philadelphia Department of Health (2020).

[8] Hoyert DL Uddin SF Miniño AM. Evaluation of the pregnancy status checkbox on the identification of maternal deaths (2020). CDC Stacks. Available online at: https://stacks.cdc.gov/view/cdc/231889 32510312

[9] Rossen LM Womack LS Hoyert DL Anderson RN Uddin SF. The impact of the pregnancy checkbox and misclassification on maternal mortality trends in the United States. Analytical and epidemiological studies, (2020). 44, 1–61. 32510309

[10] Stanczyk AB. The dynamics of US household economic circumstances around a birth. Demography. (2020) 57:1271–96. doi: 10.1007/s13524-020-00897-1, 32705567

[11] Paradies Y Ben J Denson N Elias A Priest N Pieterse A . Racism as a determinant of health: a systematic review and meta-analysis. PLoS One. (2015) 10:e0138511 doi: 10.1371/journal.pone.0138511 26398658 PMC4580597

[12] Hollenbach SJ Thornburg LL Glantz JC Hill E. Associations between historically redlined districts and racial disparities in current obstetric outcomes. JAMA Netw Open. (2021) 4:e2126707–7. doi: 10.1001/jamanetworkopen.2021.26707, 34591104 PMC8485176

[13] Burris HH Hacker MR. Birth outcome racial disparities: a result of intersecting social and environmental factors. Semin Perinatol. (2017) 41:360–6. doi: 10.1053/j.semperi.2017.07.002 28818300 PMC5657505

[14] Hamilton C Sariscsany L Waldfogel J Wimer C. Experiences of poverty around the time of a birth: a research note. Demography. (2023) 60:965–76. doi: 10.1215/00703370-10837403, 37326011

[15] Haushofer J Fehr E. On the psychology of poverty. Science. (2014) 344:862–7. doi: 10.1126/science.1232491, 24855262

[16] Bagstagli F Hagen-Zanker J Harman L Barca V Sturge G Schmidt T. Cash transfers: What does the evidence say? A rigorous review of programme impact and the role of design and implementation features. London: Overseas Development Institute (2016). 1–300.

[17] Lechler T. Personal communication with director of the mothers and infants cash coalition. (2025).

[18] Collective Impact Forum. What Is Collective Impact [Internet]. Collective Impact Forum. [cited 2025 Aug 28]. Available online at: https://collectiveimpactforum.org/what-is-collective-impact/ (Accessed August 30, 2025).

[19] Sappenfield WM Peck MG Gilbert CS Haynatzka VR Bryant T. Perinatal periods of risk: analytic preparation and phase 1 analytic methods for investigating Feto-infant mortality. Matern Child Health J. (2010) 14:838–50. doi: 10.1007/s10995-010-0625-4, 20563881

[20] Pennsylvania | UnitedForALICE. Available online at: https://www.unitedforalice.org/the-cost-of-basics/pennsylvania (Accessed August 20, 2025).

[21] Living Wage Calculator. Available online at: https://livingwage.mit.edu/ (Accessed August 18, 2025).

[22] American College of Obstetricians and Gynecologists’ Committee on Practice Bulletins—Gynecology. ACOG practice bulletin no. 200: early pregnancy loss. Obstet Gynecol. (2018) 132:e197–207. doi: 10.1097/AOG.0000000000002899 30157093

[23] Israel BA Schulz AJ Parker EA Becker AB. Review of community-based research: assessing partnership approaches to improve public health. Annu Rev Public Health. (1998) 19:173–202. doi: 10.1146/annurev.publhealth.19.1.173 9611617

[24] Wallerstein N Duran B. Community-based participatory research contributions to intervention research: the intersection of science and practice to improve health equity. Am J Public Health. (2010) 100:S40–6. doi: 10.2105/AJPH.2009.184036 20147663 PMC2837458

[25] Philadelphia 2023: State of the City. Available online at: https://pew.org/3KGH9Yt (Accessed August 25, 2025).

[26] Philadelphia Department of Public Health. Neighborhood rankings [Internet]. Philadelphia: City of Philadelphia; (2019). Available online at: https://www.phila.gov/media/20190731150103/Neighborhood-Rankings_7_31_19.pdf (Accessed August 30, 2025).

[27] World Health Organization. International statistical classification of diseases and related health problems, 10th revision. Geneva: World Health Organization (2019).

[28] Cox JL Holden JM Sagovsky R. Detection of postnatal depression: development of the 10-item Edinburgh postnatal depression scale. Br J Psychiatry. (1987) 150:782–6. doi: 10.1192/bjp.150.6.782 3651732

[29] Levis B Negeri Z Sun Y Benedetti A Thombs BD. Accuracy of the Edinburgh postnatal depression scale (EPDS) for screening to detect major depression among pregnant and postpartum women: systematic review and meta-analysis of individual participant data. BMJ. (2020) 371:m4022. doi: 10.1136/bmj.m4022 33177069 PMC7656313

[30] Rosander M Berlin A Forslund Frykedal K Barimani M. Maternal depression symptoms during the first 21 months after giving birth. Scand J Public Health. (2021) 49:606–15. doi: 10.1177/1403494820977969 33308010 PMC8512257

[31] Jacques N Mesenburg MA Matijasevich A Domingues MR Bertoldi AD Stein A . Trajectories of maternal depressive symptoms from the antenatal period to 24-months postnatal follow-up: findings from the 2015 Pelotas birth cohort. BMC Psychiatry. (2020) 20:233 doi: 10.1186/s12888-020-02533-z 32408866 PMC7222527

[32] Weinberg MK Tronick EZ Beeghly M Olson KL Kernan H Riley JM. Subsyndromal depressive symptoms and major depression in postpartum women. Am J Orthopsychiatry. (2001) 71:87–97. doi: 10.1037/0002-9432.71.1.87 11271721

[33] Meaney MJ. Perinatal maternal depressive symptoms as an issue for population health. Am J Psychiatry. (2018) 175:1084–93. doi: 10.1176/appi.ajp.2018.17091031 30068258

[34] Spitzer RL Kroenke K Williams JB Löwe B. A brief measure for assessing generalized anxiety disorder: the GAD-7. Arch Intern Med. (2006) 166:1092–7. doi: 10.1001/archinte.166.10.1092, 16717171

[35] Cohen S Kamarck T Mermelstein R. A global measure of perceived stress. J Health Soc Behav. (1983) 24:385–96. doi: 10.2307/2136404 6668417

[36] Boivin M Pérusse D Dionne G Saysset V Zoccolillo M Tarabulsy GM . The genetic-environmental etiology of parents’ perceptions and self-assessed behaviours toward their 5-month-old infants in a large twin and singleton sample. J Child Psychol Psychiatry. (2005) 46:612–30. doi: 10.1111/j.1469-7610.2004.00375.x, 15877767

[37] CFPB Financial Well-Being Scale: Scale development technical report [Internet]. (2017). Available online at: https://www.consumerfinance.gov/data-research/research-reports/financial-well-being-technical-report/ (Accessed August 20, 2025).

[38] Zahid N Blebu B Felder J McCulloch CE Chambers BD Curry VC . Economic insecurities and mental health among low-income pregnant people in the Central Valley region of California. Womens Health Issues. (2025) 35:105–15. doi: 10.1016/j.whi.2025.01.006 39979154

[39] Hager ER Quigg AM Black MM Coleman SM Heeren T Rose-Jacobs R . Development and validity of a 2-item screen to identify families at risk for food insecurity. Pediatrics. (2010) 126:e26–32. doi: 10.1542/peds.2009-3146, 20595453

[40] Kotelchuck M. The adequacy of prenatal care utilization index: its US distribution and association with low birthweight. Am J Public Health. (1994) 84:1486–9. doi: 10.2105/AJPH.84.9.1486, 8092377 PMC1615176

[41] Feldstein S Hane AA Morrison BM Huang KY. Relation of the postnatal attachment questionnaire to the attachment q-set. J Reprod Infant Psychol. (2004) 22:111–21. doi: 10.1080/0264683042000205972

[42] Condon JT Corkindale CJ. The assessment of parent-to-infant attachment: development of a self-report questionnaire instrument. J Reprod Infant Psychol. (1998) 16:57–76. doi: 10.1080/02646839808404558

[43] Matthey S. Calculating clinically significant change in postnatal depression studies using the Edinburgh postnatal depression scale. J Affect Disord. (2004) 78:269–72. doi: 10.1016/S0165-0327(02)00313-0 15013253

[44] Sample Size Software | Power Analysis Software | PASS | NCSS.com [Internet]. [cited 2024 Mar 14]. Available online at: https://www.ncss.com/software/pass/

[45] Arcaya MC Schnake-Mahl A Binet A Simpson S Church MS Gavin V . Community change and resident needs: designing a participatory action research study in metropolitan Boston. Health Place. (2018) 52:221–30. doi: 10.1016/j.healthplace.2018.05.014 30015179

[46] Bach M Jordan S Hartung S Santos-Hövener C Wright MT. Participatory epidemiology: the contribution of participatory research to epidemiology. Emerg Themes Epidemiol. (2017) 14:1–15. doi: 10.1186/s12982-017-0056-4 28203262 PMC5301332

[47] Wallerstein N Oetzel JG Duran B Magarati M Pearson C Belone L . Culture-centeredness in community-based participatory research: contributions to health education intervention research. Health Educ Res. (2019) 34:372–88. doi: 10.1093/her/cyz021, 31237937 PMC6646947

[48] Woodman D Tyler D. Participatory approaches to longitudinal research with young people. Youth Stud Aust. (2007) 26:20–6. doi: 10.3316/informit.464612545536382

[49] Alio AP Dillion T Hartman S Johnson T Turner S Bullock S . A community collaborative for the exploration of local factors affecting Black mothers’ experiences with perinatal care. Matern Child Health J. (2022) 26:751–60. doi: 10.1007/s10995-022-03422-5, 35316456 PMC8938641

[50] Mendenhall R Henderson L Scott B Butler L Turi KN Greenlee A . Involving urban single low-income African American mothers in genomic research: giving voice to how place matters in health disparities and prevention strategies. Family Med Primary Care. (2020) 4:148. doi: 10.29011/2688-7460.100048, 35373191 PMC8970351

[51] Letiecq BL Williams JM Vesely CK Lee JS. Publicly housed Black mothers’ experiences of structural racism in their everyday lives. J Marriage Fam. (2023) 85:701–22. doi: 10.1111/jomf.12908

[52] MacEntee K Burkholder C Schwab-Cartas J, editors. What’s a Cellphilm? [Internet]. Rotterdam: SensePublishers; (2016)

[53] Tsang KK. Photovoice data analysis: critical approach, phenomenological approach, and beyond. Beijing Int Rev Educ. (2020) 2:136–52. doi: 10.1163/25902539-00201009

[54] Wang CC Yi WK Tao ZW Carovano K. Photovoice as a participatory health promotion strategy. Health Promot Int. (1998) 13:75–86. doi: 10.1093/heapro/13.1.75

[55] Jackson FM Saran AR Ricks S Essien J Klein K Roberts D . Save 100 babies©: engaging communities for just and equitable birth outcomes through photovoice and appreciative inquiry. Matern Child Health J. (2014) 18:1786–94. doi: 10.1007/s10995-014-1436-9, 24474593

[56] Comas-Díaz L. Latino healing: the integration of ethnic psychology into psychotherapy. Psychother Theory Res Pract Train. (2006) 43:436–53. doi: 10.1037/0033-3204.43.4.436, 22122135

[57] Rennick-Egglestone S Ramsay A McGranahan R Llewellyn-Beardsley J Hui A Pollock K . The impact of mental health recovery narratives on recipients experiencing mental health problems: qualitative analysis and change model. PLoS One. (2019) 14:e0226201. doi: 10.1371/journal.pone.0226201 31834902 PMC6910821

[58] Schensul JJ LeCompte MD. Essential ethnographic methods: A mixed methods approach. Rowman Altamira. New York, NY: Bloomsbury Publishing Inc. (2013). 387 p.

[59] Hanna M Shaefer HL Fogle H Khaldun JS McWeeny W Richardson O . Scaling up prenatal and infant cash prescriptions to eradicate deep infant poverty in the United States [internet]. Washington (DC): Brookings Institution (2024).

[60] Karasek D Williams JC Taylor MA De La Cruz MM Arteaga S Bell S . Designing the first pregnancy guaranteed income program in the United States: qualitative needs assessment and human-centered design to develop the abundant birth project. JMIR Form Res. (2025) 9:e60829. doi: 10.2196/60829, 39869889 PMC11811656

[61] Hanna M Shaefer HL Finegood E Agarwal S Zamani-Hank Y LaChance J. Hardship and hope: the relationship between unconditional prenatal and infant cash transfers, economic stability, and maternal mental health and well-being. Am J Public Health. (2025) 115:2020–29. doi: 10.2105/AJPH.2025.308244 40934444 PMC12614019

[62] Magnuson KA Duncan GJ Yoshikawa H Yoo P Han S Gennetian LA . Effects of unconditional cash transfers on family processes and wellbeing among mothers with low incomes. Nat Commun. (2025) doi: 10.1038/s41467-025-62438-x PMC12350725 40804074

[63] Kovski N Pilkauskas NV Michelmore K Shaefer HL. Unconditional cash transfers and mental health symptoms among parents with low incomes: evidence from the 2021 child tax credit. SSM. (2023) 22:101420. doi: 10.1016/j.ssmph.2023.101420, 37151915 PMC10148983

[64] Schmidt L Shore-Sheppard L Watson T. The effect of safety net generosity on maternal mental health and risky health behaviors. J Policy Anal Manage. (2023) 42:706–36. doi: 10.1002/pam.22481

[65] Enns JE Nickel NC Chartier M Chateau D Campbell R Phillips-Beck W . An unconditional prenatal income supplement is associated with improved birth and early childhood outcomes among first nations children in Manitoba, Canada: a population-based cohort study. BMC Pregnancy Childbirth. (2021) 21:1–11. doi: 10.1186/s12884-021-03782-w 33879074 PMC8059008

[66] Andrews KG Martin MW Shenberger E Pereira S Fink G McConnell M. Financial support to Medicaid-eligible mothers increases caregiving for preterm infants. Matern Child Health J. (2020) 24:587–600. doi: 10.1007/s10995-020-02905-7, 32277384

[67] Sperber JF Gennetian LA Hart ER Kunin-Batson A Magnuson K Duncan GJ . Unconditional cash transfers and maternal assessments of children’s health, nutrition, and sleep: a randomized clinical trial. JAMA Netw Open. (2023) 6:e2335237–e2335237. doi: 10.1001/jamanetworkopen.2023.35237 37773497 PMC10543132

[68] Hart ER Gennetian LA Sperber JF Penalva R Magnuson K Duncan GJ . The effect of unconditional cash transfers on maternal assessments of children’s early language and socioemotional development: experimental evidence from US families residing in poverty. Dev Psychol. (2024) 60:2290–305. doi: 10.1037/dev0001824, 39172428 PMC11906382

[69] Egan-Dailey S Gennetian LA Magnuson K Duncan GJ Yoshikawa H Fox NA . Child-directed speech in a large sample of US mothers with low income. Child Dev. (2024) 95:2045–61. doi: 10.1111/cdev.14139, 39073390 PMC12145882

[70] Calancie L Batdorf-Barnes A Verbiest S White N Lich KH Corbie G . Practical approaches for promoting health equity in communities. Matern Child Health J. (2022) 26:82–7. doi: 10.1007/s10995-022-03456-9, 35920955 PMC9482601

[71] Wittkowski A Vatter S Muhinyi A Garrett C Henderson M. Measuring bonding or attachment in the parent-infant-relationship: a systematic review of parent-report assessment measures, their psychometric properties and clinical utility. Clin Psychol Rev. (2020) 82:101906. doi: 10.1016/j.cpr.2020.101906, 32977111 PMC7695805

[72] Condon J. Assessing attachment, a work in progress: to look, to listen or both? J Reprod Infant Psychol. (2012) 30:1–4. doi: 10.1080/02646838.2012.681966

[73] Gennetian LA Duncan G Fox NA Magnuson K Halpern-Meekin S Noble KG Unconditional cash and family investments in infants: Evidence from a large-scale cash transfer experiment in the US National Bureau of Economic Research. Cambridge, MA: National Bureau of Economic Research (2022). Available online at: https://www.nber.org/papers/w30379

